# Fulminant Hepatic Failure Following Initiation of Abiraterone in Metastatic Prostate Cancer: A Fatal Adverse Drug Reaction

**DOI:** 10.1155/crhe/8134585

**Published:** 2026-06-10

**Authors:** Emma Tilma, Esben Bolvig Mark, Asbjørn Mohr Drewes

**Affiliations:** ^1^ Department of Internal Medicine, Aalborg University Hospital, Højtoftevej 2, Thisted, 7700, Denmark, aalborguh.rn.dk; ^2^ Thisted Research Unit, Aalborg University Hospital, Thisted, Højtoftevej 2, Thisted, 7700, Denmark; ^3^ Mech-Sense, Department of Gastroenterology and Hepatology, Aalborg University Hospital, Aalborg, Mølleparkvej 4, Aalborg, 9000, Denmark; ^4^ Department of Clinical Medicine, Aalborg University, Aalborg, Selma Lagerløfs Vej 249, Gistrup, 9260, Denmark

**Keywords:** abiraterone, case report, drug-induced liver injury, fulminant hepatic failure, hepatotoxicity

## Abstract

**Background:**

Abiraterone acetate is widely used in metastatic castration‐resistant prostate cancer and is generally considered safe. Hepatotoxicity is a known adverse effect, but fulminant liver failure remains a rare and potentially fatal complication.

**Case presentation:**

An 80‐year‐old man with metastatic prostate cancer was admitted with high‐grade fever and general deterioration three weeks after initiation of abiraterone in addition to ongoing androgen deprivation therapy. He presented with jaundice, hypotension, and altered mental status. Laboratory investigations revealed severe acute liver injury with markedly elevated transaminases, coagulopathy, hyperbilirubinemia, acute kidney injury, metabolic acidosis, and hypoglycemia. Computed tomography demonstrated marked periportal hepatic edema and mild hilar lymphadenopathy, consistent with severe acute liver injury. No focal hepatic lesions or malignancy were identified. Despite discontinuation of abiraterone and supportive care, there was a progression to multiorgan failure, and the patient died within 48 h of admission. Postmortem investigations excluded acute hepatitis A and cytomegalovirus infection. Epstein–Barr virus serology was consistent with past infection, excluding acute infection. Although histological confirmation was not obtained, the clinical course and temporal association were highly suggestive of drug‐induced liver injury.

**Conclusion:**

This case highlights a rare but catastrophic adverse reaction to abiraterone. Regular monitoring of liver function and early consideration of hepatotoxicity in patients presenting with systemic symptoms during treatment are essential.

## 1. Introduction

Abiraterone acetate (Zytiga, Janssen Pharmaceuticals) is a CYP17A1 inhibitor commonly used in the treatment of metastatic castration‐resistant prostate cancer. Although generally well tolerated, hepatotoxicity has been reported, with elevations in liver enzymes occurring in up to 13%–15% of treated individuals [1, 2]. Severe hepatic injury, including fulminant liver failure, has only rarely been reported, but remains a life‐threatening complication of the therapy [1, 3, 4]. Current prescribing information recommends close monitoring of liver function during therapy, particularly in the early treatment phase [5]. We present a fatal case of acute liver failure presenting shortly after initiation of abiraterone acetate therapy.

## 2. Case Presentation

An 80‐year‐old man with diverticulosis, ischemic heart disease, and prostate cancer with metastatic involvement of mediastinal and hilar lymph nodes and a sclerotic lesion in the L3 vertebral body was admitted on November 17^th^, 2025, due to fever and general deterioration.

He had undergone a radical prostatectomy in 2011, followed by salvage radiotherapy and intermittent androgen deprivation therapy due to biochemical recurrence of the malignancy. In October 2025, radiological staging showed disease progression with pathological lymph nodes in the mediastinum, both pulmonary hila, and the retroperitoneum, as well as a bone metastasis at L3. Prostate‐specific antigen increased to 49.7 ng/mL during bicalutamide treatment, and treatment was switched to leuprolide and abiraterone combined with prednisolone.

At the first follow‐up on November 10^th^, approximately four weeks after initiation of abiraterone, prostate‐specific antigen was decreasing, and testosterone was < 0.12 nmol/L, consistent with castrate levels. Liver enzymes were elevated (alanine aminotransferase 137 U/L on November 13), supporting that the hepatic injury had begun before the patient’s acute decline, followed by rapid progression to fulminant hepatic failure within days.

On November 15th, the patient developed nausea, abdominal pain, profuse sweating, and fever. A temperature of 39.4°C was measured at home the day prior to admission. At presentation, the rectal temperature was 40.5°C. Clinical examination revealed jaundice, hypotension, confusion, and diffuse abdominal tenderness.

Initial laboratory investigations revealed striking evidence of acute hepatic collapse and emerging coagulopathy, as outlined in Table 1. Differential blood count showed eosinophilia, supporting a possible immune‐mediated drug‐induced liver injury.

**TABLE 1:** Key laboratory values during the hospital course.

Parameter	Approx. 4 weeks after initiation of abiraterone (Nov 13)	Admission (Nov 17)	Day 2 (Nov 18)
ALT (U/L)	137	1080	> 2000
Bilirubin (μmol/L)	8	30	45
INR	1.1	2.1	3.0
LDH (U/L)	315	1277	—
Creatinine (μmol/L)	96	156	209
CRP (mg/L)	6.2	166	242
Eosinophils (n/L)	—	0,54 × 10^9^	—
Lactate (mmol/L)	—	—	15
Blood glucose (mmol/L)	7.6	8.0	1.0
Total plasma calcium (mmol/L)	2.31	2.0	0.99 (ionized calcium)

*Note:* ALT, alanine aminotransferase; LDH, lactate dehydrogenase.

Abbreviation: INR, international normalized ratio.

Computed tomography of the abdomen demonstrated marked periportal hepatic edema and mild hilar lymphadenopathy, without focal hepatic lesions or signs of tumor progression (Figure 1). Additional findings included inflammatory changes around the gallbladder and pancreas, ascites, and bilateral pleural effusions.

**FIGURE 1:**
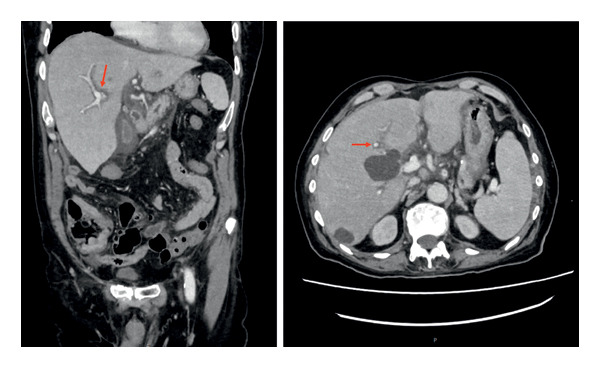
Contrast‐enhanced computed tomography of the abdomen showing periportal edema (arrow) and diffuse hepatic parenchymal changes on coronal (left) and axial (right) images, consistent with severe acute liver injury.

The patient was initially treated with piperacillin/tazobactam for presumed sepsis, aggressive intravenous fluids for hypotension and acute kidney injury, and intravenous proton pump inhibitors. Within 24 h, his clinical status declined precipitously, marked by profound hypoglycemia, worsening of the biochemical instability, and rapidly escalating indicators of hepatic failure (see Table 1). He developed acute respiratory failure requiring high‐flow oxygen therapy and developed severe metabolic acidosis with a lactate of 15 mmol/L, consistent with fulminant liver failure and multiorgan dysfunction.

Abiraterone and all potentially hepatotoxic drugs were discontinued. Hepatitis A and E serology, cytomegalovirus polymerase chain reaction, and Epstein–Barr virus (EBV) testing were obtained. Vitamin K was administered, and supportive care was provided.

Despite treatment, the patient rapidly deteriorated and died on 19 November 2025.

Subsequent infection screening showed negative results for hepatitis A (IgM and IgG) and cytomegalovirus DNA. EBV testing was consistent with past infection with positive viral capsid antigen (VCA) IgG and Epstein–Barr nuclear antigen IgG, and negative VCA IgM, thereby excluding acute EBV infection.

## 3. Discussion

This case represents a severe and likely abiraterone‐induced acute liver failure. Abiraterone is metabolized predominantly via hepatic CYP450 enzymes, and hepatotoxicity is a recognized adverse effect [1, 5]. A strong temporal relationship between drug initiation and symptom onset, combined with the exclusion of hepatitis A, EBV, and cytomegalovirus infection, and the absence of radiological malignancy, strongly supports a diagnosis of drug‐induced liver injury.

A retrospective causality assessment using the Roussel Uclaf Causality Assessment Method (RUCAM) (Council for International Organizations of Medical Sciences [CIOMS]) [6] yielded a score of 6, indicating a probable association between abiraterone and the observed liver injury (see Table 2). This objective assessment strengthens the likelihood of drug‐induced hepatotoxicity, although some competing causes could not be fully excluded.

**TABLE 2:** RUCAM (CIOMS) causality assessment for drug‐induced liver injury.

Parameter	Score
Time to onset	+2
Course after cessation	0
Risk factors (age > 55)	+1
Concomitant drugs	0
Exclusion of other causes	+1
Known hepatotoxicity	+2
Rechallenge	0
Total score	**6 (probable)**

*Note:* Bold value indicates the final RUCAM assessment result (total score 6, corresponding to a probable drug‐induced liver injury).

Abbreviation: RUCAM, Roussel Uclaf Causality Assessment Method.

Other causes of acute liver failure were considered. Infectious etiologies, including hepatitis A, cytomegalovirus, and EBV, were excluded. Imaging showed no evidence of biliary obstruction or hepatic malignancy. There were no clinical or microbiological findings to support sepsis or ischemic hepatitis. Autoimmune hepatitis (ANA, ASMA, and IgG) and other viral hepatitis (HBV and HEV) were not formally assessed, which represents a limitation. However, these etiologies were considered less likely given the clinical presentation, temporal relationship to drug initiation, and the rapid progression to fulminant hepatic failure. No other hepatotoxic medications were identified as likely contributors.

The presence of eosinophilia further supports this diagnosis, as eosinophilia is a recognized feature of immune‐mediated and idiosyncratic drug‐induced liver injury [7].

Fatal cases of abiraterone‐associated acute liver failure have previously been reported, supporting a causal relationship between treatment and fulminant hepatic injury [3, 4]. Observational data further demonstrate an increased risk of hospitalization due to adverse events, including hepatotoxicity, among patients treated with abiraterone compared to androgen receptor pathway inhibitors [8]. Real‐world cohort data additionally show that liver enzyme elevations typically occur early during treatment and may progress rapidly in susceptible individuals [2].

Although early discontinuation of abiraterone is recommended when hepatotoxicity is suspected, mortality in fulminant drug‐induced liver injury remains high, particularly in elderly and comorbid patients. Current prescribing guidance emphasizes the need for close monitoring of liver function, especially during the initial phase of therapy [1, 5]. Although most abnormalities are mild and reversible, severe hepatic injury and fulminant liver failure, as demonstrated in this case, remain rare but life‐threatening complications.

## 4. Conclusion

This case illustrates a rare but catastrophic adverse reaction to abiraterone, resulting in fulminant hepatic failure and rapid multiorgan deterioration shortly after treatment initiation. Clinicians should maintain a high index of suspicion for early signs of hepatotoxicity, including fatigue, fever, and abdominal discomfort, and ensure close monitoring of liver function during the initial weeks of therapy.

## 5. Patient Perspective

A patient perspective was not available due to the rapid clinical deterioration and fatal outcome.

## Author Contributions

Conceptualization: Emma Tilma.

Data collection and investigation: Emma Tilma.

Formal analysis: Emma Tilma.

Writing–original draft: Emma Tilma.

Writing–review and editing: Emma Tilma, Esben Bolvig Mark, and Asbjørn Mohr Drewes.

Supervision: Esben Bolvig Mark and Asbjørn Mohr Drewes.

## Funding

No funding or sponsorship was received for this study or the publication of this article.

## Disclosure

All authors read and approved the final manuscript.

## Ethics Statement

This case report is based on a retrospective review of a single patient case.

According to Danish legislation and institutional policy, ethics committee approval is not required for case reports based on anonymized clinical information.

The study was conducted in accordance with the Helsinki Declaration (1964) and its later amendments. Consent for publication of anonymized clinical details was obtained from the patient’s next of kin. The study was registered at the regional research database with ID no: K2025‐241 as per local legislation.

## Consent

Please see the Ethics Statement.

## Conflicts of Interest

The authors declare no conflicts of interest.

## Data Availability

Data are available from the corresponding author upon reasonable request. Due to patient confidentiality and privacy regulations, data are not publicly available.
